# Collaborative Impact of Compost and Beneficial Rhizobacteria on Soil Properties, Physiological Attributes, and Productivity of Wheat Subjected to Deficit Irrigation in Salt Affected Soil

**DOI:** 10.3390/plants11070877

**Published:** 2022-03-25

**Authors:** Alaa El-Dein Omara, Emad M. Hafez, Hany S. Osman, Emadeldeen Rashwan, Mohamed A. A. El-Said, Khadiga Alharbi, Diaa Abd El-Moneim, Salah M. Gowayed

**Affiliations:** 1 Department of Microbiology, Soils, Water and Environment Research Institute, Agricultural Research Center, Giza 12112, Egypt; alaa.omara@yahoo.com; 2 Department of Agronomy, Faculty of Agriculture, Kafrelsheikh University, Kafr El-Sheikh 33516, Egypt; 3 Department of Agricultural Botany, Faculty of Agriculture, Ain Shams University, Hadayek Shubra, Cairo 11241, Egypt; 4 Agronomy Department, Faculty of Agriculture, Tanta University, Tanta 31527, Egypt; emad.rashwan@agr.tanta.edu.eg; 5 Department of Agronomy, Faculty of Agriculture, Al-Azhar University, Assiut 71524, Egypt; mohamedel-saied.49@azhar.edu.eg; 6 Department of Biology, College of Science, Princess Nourah Bint Abdulrahman University, Riyadh 11671, Saudi Arabia; 7 Department of Plant Production (Genetic Branch), Faculty of Environmental Agricultural Sciences, Arish University, El-Arish 45511, Egypt; dabdelmoniem@aru.edu.eg; 8 Department of Botany, Faculty of Agriculture, Suez Canal University, Ismailia 41522, Egypt; salahgowed@yahoo.com

**Keywords:** antioxidant, compost, deficit irrigation, drought, plant growth promoting rhizobacteria, sustainability, *Triticum aestivum*, yield

## Abstract

Plant growth and crop productivity under unfavorable environmental challenges require a unique strategy to scavenge the severely negative impacts of these challenges such as soil salinity and water stress. Compost and plant growth-promoting rhizobacteria (PGPR) have many beneficial impacts, particularly in plants exposed to different types of stress. Therefore, a field experiment during two successive seasons was conducted to investigate the impact of compost and PGPR either separately or in a combination on exchangeable sodium percentage (ESP), soil enzymes (urease and dehydrogenase), wheat physiology, antioxidant defense system, growth, and productivity under deficient irrigation and soil salinity conditions. Our findings showed that exposure of wheat plants to deficit irrigation in salt-affected soil inhibited wheat growth and development, and eventually reduced crop productivity. However, these injurious impacts were diminished after soil amendment using the combined application of compost and PGPR. This combined application enhanced soil urease and dehydrogenase, ion selectivity, chlorophylls, carotenoids, stomatal conductance, and the relative water content (RWC) whilst reducing ESP, proline content, which eventually increased the yield-related traits of wheat plants under deficient irrigation conditions. Moreover, the coupled application of compost and PGPR reduced the uptake of Na and resulted in an increment in superoxide dismutase (SOD), catalase (CAT), and peroxidase (POX) activities that lessened oxidative damage and improved the nutrient uptake (N, P, and K) of deficiently irrigated wheat plants under soil salinity. It was concluded that to protect wheat plants from environmental stressors, such as water stress and soil salinity, co-application of compost with PGPR was found to be effective.

## 1. Introduction

Wheat (*Triticum aestivum* L.) is deemed the major crop in Egypt with a total cultivation area of nearly 50%, which is planted by roughly 90% of farmers. Globally, there is 778 Mt of wheat produced annually which means 35% of the world’s total food grains [1]. Wheat is considered the second source of calories and the largest protein source after the rice crop [2]. However, the production of the wheat crop is very insufficient owing to various reasons including the high use of chemical fertilizers and exposure to abiotic stresses such as soil salinity and water stress which harmfully impacts soil health, plant growth that results in 50% crop reduction [3,4]. Many field crops suffer from soil salinity and water stress; therefore it is crucial to explore alternative ways which mitigate the detrimental impact of salinity and drought on wheat growth and productivity [5,6]. Consequently, sustainable wheat production and exploring new techniques and strategies to combat the negative impacts of salinity and water stress alongside increasing wheat production are important [7,8].

In the Mediterranean zone, salt-affected soil has been stated to have an adverse effect on soil health and plant development, influencing 25 to 30% of plant production [9]. Furthermore, salt-affected soil is a main abiotic stressor that impairs productivity in agricultural soil [10]. Salt-affected soil of cultivated land is predicted to have destructive impacts worldwide, causing 30% of wasted land over the coming 25 years and 50% over the coming 50 years [11]. Currently, 50% of the global total arable area is subjected to salt stressors, producing a loss of nearly USD 13 billion [12]. Furthermore, salt stress is an intricate process that has harmful impacts on plant performance and development [13]. Consequently, Na^+^ impedes metabolic function, especially; enzymes involved in photosynthesis or osmotic disturbance results in lessening in osmotic regulation, specific-ion toxicity, and the production of reactive oxygen species (ROS), plant development, water uptake, and nutrient uptake [14]. Therefore, it is imperative to investigate agronomically sound practices to combat the destructive impact of salt stress by several mechanisms, i.e., choosing the appropriate cultivar in saline conditions is the major imperative factor in the success of the cultivation process and attaining an economic return, soil amendments, and biological products.

In the arid and semi-arid regions also, water stress is a serious phenomenon devastating affecting agricultural production [15]. Climate changes scenarios expect that precipitation will decrement and the frequency of water stress will increment causing an actual menace to food security, highlighting the need for promising crops that tolerate these harsh circumstances [16]. Under the choice for water stress regimes or rainfed regions, economic crops give less yields or cannot be cultivated owing to a limitation of the root architecture and decline in leaf elongation rate, which finally affecting plant phenology [17]. Water stress decreases the morphological properties and physiological processes, reduces seed germination, seedling establishment, flowering, and seed development. Moreover, water stress causes oxidative stress and diminishes relative water content and nutrient uptake [18,19]. It is therefore important to explore a technique to enhance crops’ water stress tolerance.

At the start of the 21st century, mankind had to answer numerous issues such as those in association with variable abiotic conditions, such as water stress or soil salinity [20]. Plants are continuously subjected to changeable abiotic factors, and while these plants are unable to escape from the adverse circumstances, they should either tolerate them or die. In recent decades, scientific research has begun focusing on developing natural and safe substances to enhance plant development, growth, crop productivity, and quality as well as combat harsh environmental conditions [21,22]. Compost and plant growth-promoting rhizobacteria are more ecologically friendly; enhance uptake, absorption, and translocation of nutrients to plant tissues during critical stages of plant growth and development under abiotic stress [23,24].

Residues of plant wastes such as straws, stoves, and green leaves are considered raw materials for compost production. The traditional methods to get rid of these residues are a source of environmental pollution by open-field burning causes air pollution. Compost is an organic amendment that has been decomposed through composting (residues of plant wastes) [25]. Recently, organic conditioner applications such as compost to semi-arid zones can be used to combat the harmful impacts of soil salinity as well as water stress [25,26]. Consequently, compost application is considered a successful agricultural strategy that improves the soil structure [27] and water holding capacity [28] as well as augments the soil nutrient availability [29], soil enzyme activity [30], and soil physicochemical characteristics under harsh environmental conditions [31]. The application of compost as a soil amendment is considered a cost-effective and eco-friendly method [31,32]. 

Rhizobacteria are presented in the plant rhizosphere have potential positive impacts on root and plant growth, those rhizobacteria are called plant growth-promoting rhizobacteria (PGPR) [29]. The inoculation with beneficial microbes is an eco-friendly and sustainable strategy for improving crop yield under environmental stressors [19]. The PGPR species have multiple modes of action [33,34]. Numerous species of rhizobacteria such as *Pseudomonas*, *Azospirillum*, *Azotobacter*, *Klebsiella*, *Enterobacter*, *Alcaligenes*, *Arthrobacter*, *Burkholderia*, *Bacillus*, and *Serratia* have been examined to increase the plant growth and development under soil salinity and water stress which causes a harmful impact on the plants’ root growth due to an increase in ethylene production [35]. There are particular salinity and drought-tolerant PGPR (*Azospirillum lipoferum SP2*, *Bacillus coagulans NCAIM B.01123*, *Bacillus circulance NCAIM B.02324*, *Bacillus subtilis MF497446,* etc.) which have ACC-deaminase activity that assists in decreasing ethylene stress on roots [36]. ACC-deaminase activity manipulates phytohormonal signaling and releases an enzyme 1 aminocyclopropane-1-carboxylic acid (ACC)-deaminase, production of exopolysaccharide (EPS), and increases the soil enzymes activity that has the ability to ameliorate the harmful impact of water and salt stress on root plant and development that converts ethylene into ammonia which eventually increases the water holding capacity [37]. PGPR can encourage plant growth and development under water and salt stresses through other mechanisms including the production of different phytohormones (gibberellic acid, auxins, cytokinins, and so on), etc., as well as has the potential to biological nitrogen fixation (BNF) through a symbiotic relationship between Rhizobia and plants [37].

Although, the application of compost or PGPR has the potential to improve soil quality that assists the cultivated plants to ameliorate the adverse effects of a variety of abiotic conditions (drought and soil salinity). However, the combined effects of compost and PGPR on soil characteristics and enzymes in salt-affected soils are untested. As a result, we hypothesized that adding compost and PGPR to salt-affected soil would improve wheat growth and productivity.

## 2. Results

### 2.1. Effect of Soil Treatments on Soil Exchangeable Na Percentage, Soil Enzymes, Ion Selectivity and Photosynthetic Pigment Contents under Abiotic Stress

Subjecting wheat plants to deficit irrigation significantly increased the exchangeable sodium percentage (ESP) in salt-affected soil compared to well-irrigated soil, which recorded an increase in ESP by 32.1% over well-irrigated soil (Table 1). Soil amendment with compost was better than PGPR application in decreasing the ESP levels. Regarding the comparison with control of regular irrigation, the application of compost and PGPR reduced the ESP by 33.9% and 17.8%, respectively, under regular irrigation, and 23.4% and 11.3% under deficit irrigation. The most effective application in reducing the ESP levels was the combined application (Compost + PGPR) either under regular or deficit irrigation, which recorded a 47% decrease than the control of the regular irrigation, and a 30% decrease than the control of the deficit irrigation. Additionally, the combined application reduced the ESP percentage under deficit irrigation by 7.9% than the control of regular irrigation (Table 1).

During the 2019/2020 and 2020/2021 growing seasons, the activity of urease and dehydrogenase in the soil grown with wheat plants decreased significantly under deficit irrigation compared to regular watering in salt-affected soil. Urease activity decreased by 25.9%, while dehydrogenase activity decreased by 38.2% under deficit irrigation (Table 1). The application of compost and PGPR individually or in a combination with each other considerably increased the activity of soil urease and dehydrogenase during both seasons, hence reducing the negative impact of deficit irrigation on the crop. Under both irrigation levels, the individual application of PGPR had a greater influence on the increase in soil urease and dehydrogenase activity than the application of compost. When plants were treated with the compost and PGPR, the maximum activity of soil enzymes was observed, regardless of the amount of irrigation applied to the plants (Table 1).

The highest concentration of Na ions and the lowest concentration of K ions, and also the lowest K/Na ratio were found in the leaves of wheat grown under deficit irrigation during the 2019/2020 and 2020/2021 growing seasons (Table 2). In this regard, the application of deficit irrigation increased Na content in wheat leaves by 45.3% over the control of regular irrigation, whereas K content decreased by 24.9% under the same condition. Compost application was more efficient than PGPR application in reducing the Na content and increasing the K content in comparison with control. Regardless of irrigation intensity, the highest K and lowest Na concentrations were found when plants were treated with a synergistic application of compost and PGPR over both seasons (Table 2). 

### 2.2. Effect of Soil Treatments on Physiological Attributes under Abiotic Stress

**Figure 1:**
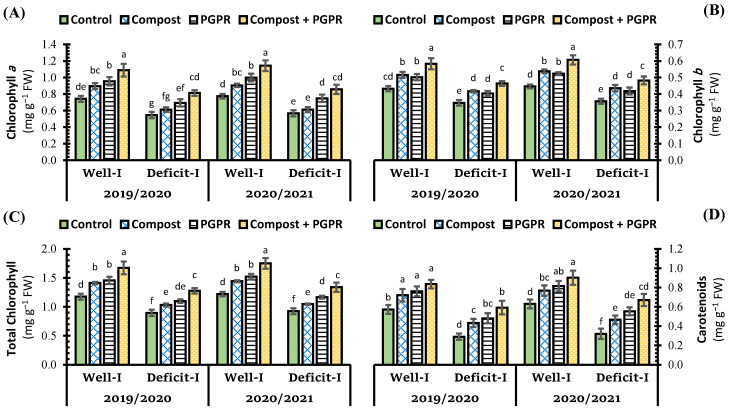
Influence of soil amendment with compost and plant growth-promoting rhizobacteria (PGPR) on the concentration of photosynthetic pigments (**A**) chlorophyll *a*, (**B**) chlorophyll *b*, (**C**) total chlorophyll, and (**D**) carotenoids in the leaves of wheat plants grown in salt-affected soil and deficit irrigation in contrast to well irrigation. The error bars present the values of standard deviation. According to Duncan’s multiple range test, the same lower-case letters above columns are not significantly different (*p* < 0.05).

The concentrations of chlorophyll *a*, *b*, and carotenoids in the leaves of the wheat plant were negatively affected by deficit irrigation (control (−)) compared to regular irrigation (control (+)) treatment, which markedly decreased the concentration of chlorophyll *a*, *b*, and total chlorophylls by 26.5%, 19.8%, and 24.0%, respectively (Figure 1), which indicates that chlorophyll *a* is more sensitive than chlorophyll *b* to drought stress. Furthermore, the addition of compost or PGPR, either alone or in combination, mitigated the negative impacts of deficit irrigation while simultaneously increasing the concentration of photosynthetic pigments in wheat leaves. Wheat plants inoculated with PGPR had a higher content of chlorophyll *a*, total chlorophylls, and carotenoids than recorded from the soil amendment with compost. In contrast to the control (+) treatment of regular irrigation, the highest increase in Chl *a*, Chl *b*, total chlorophyll, and carotenoids over the control (+) was remarked with the dual application of compost (+) PGPR, which increased the percentage over the control (+) by 46.7%, 34.8%, 42.3%, and 45.9% for the regular irrigation condition, whereas under the deficit irrigation conditions, the percentage of increments over the same positive control were 9.6%, 7.2%, 8.7%, and 3.3%, respectively (Figure 1). 

**Figure 2:**
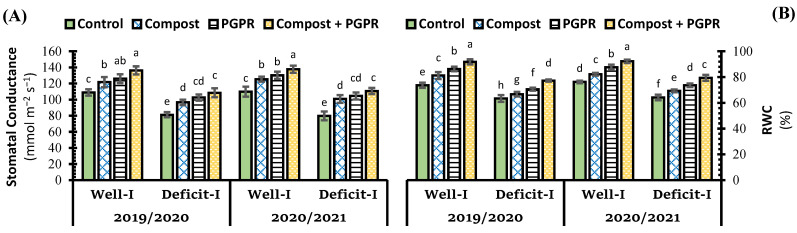
Influence of soil amendment with compost and plant growth-promoting rhizobacteria (PGPR) on (**A**) stomatal conductance, and (**B**) relative water content (RWC) in the leaves of wheat plants grown in salt-affected soil and deficit irrigation in contrast to well irrigation. The error bars present the values of standard deviation. According to Duncan’s multiple range test, the same lower-case letters above columns are not significantly different (*p* < 0.05).

Subjecting wheat plants to deficit irrigation in salt-affected soil resulted in a reduction in the parameters of water relations, i.e., stomatal conductance (gs) and relative water content (RWC) (Figure 2). Deficit irrigation led to reducing stomatal conductance by 25.4%, while RWC reduced by 14.1% below the control^+^ treatment. Individual application of PGPR enhanced the RWC and gs of wheat leaves by 17.2% and 15.8%, respectively, whereas compost treatment had a less effect, which recorded a 10.2% and 11.8% increase over control (+), respectively. The largest RWC was reported when compost and PGPR were applied concurrently, leading to a 24.6% and 22.1% increase over control (+) and control (−), respectively (Figure 2B). Not only the combined application recorded the highest RWC, but also increased its level under deficit irrigation conditions by 4.9% over control (+). Similarly, stomatal conductance followed a similar trend (Figure 2A).

### 2.3. Effect of Soil Treatments on Oxidative Stress Indicators and Antioxidant System in Wheat Leaves under Abiotic Stress

**Figure 3:**
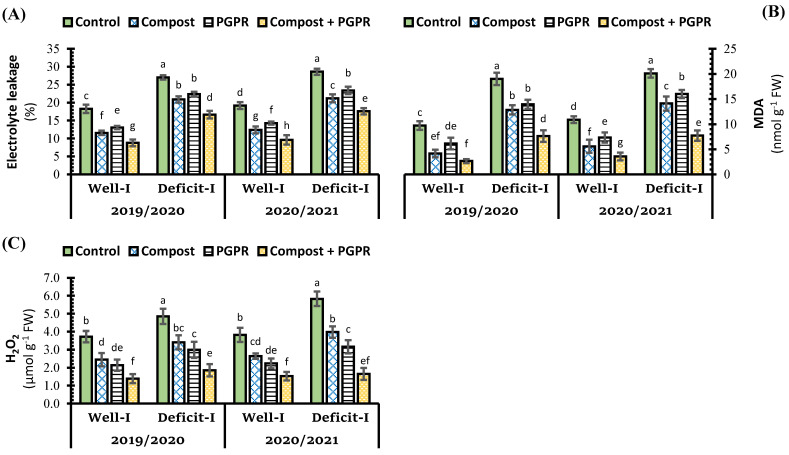
Influence of soil amendment with compost and plant growth-promoting rhizobacteria (PGPR) on (**A**) electrolyte leakage percentage, (**B**) malondialdehyde (MDA) concentration, and (**C**) hydrogen peroxide (H_2_O_2_) concentration in the leaves of wheat plants grown in salt-affected soil and deficit irrigation in contrast to well irrigation. The error bars present the values of standard deviation. According to Duncan’s multiple range test, the same lower-case letters above columns are not significantly different (*p* < 0.05).

In both seasons, deficit irrigation positively affects stress indicators in wheat plants, which recorded a significant increase over control (+) in the percentage of EL by 47.9%, and the concentration of MDA by 95.5%, and H_2_O_2_ 30.4%, respectively (Figure 3). Levels of EL%, MDA, and H_2_O_2_ in wheat plants were reduced under all intervals, either for individual application or combined application with compost and PGPR. The sole application with compost was more significant than PGPR application in reducing the levels of EL% and MDA, whereas PGPR application was more effective than compost application in minimizing the levels of formed H_2_O_2_. Under all irrigation treatments, co-application of compost and PGPR was found to be the most successful at lowering the levels of stress indicators. Under regular irrigation, the combined treatment reduced EL% by 52%, below control (+), whereas under deficit irrigation, the same combined treatment reduced the EL% by 38.4% below control (−). The concentrations of MDA reduced under the effect of the combined application by 72.8% and 59.7% below their own controls (+ and −), respectively. 

**Figure 4:**
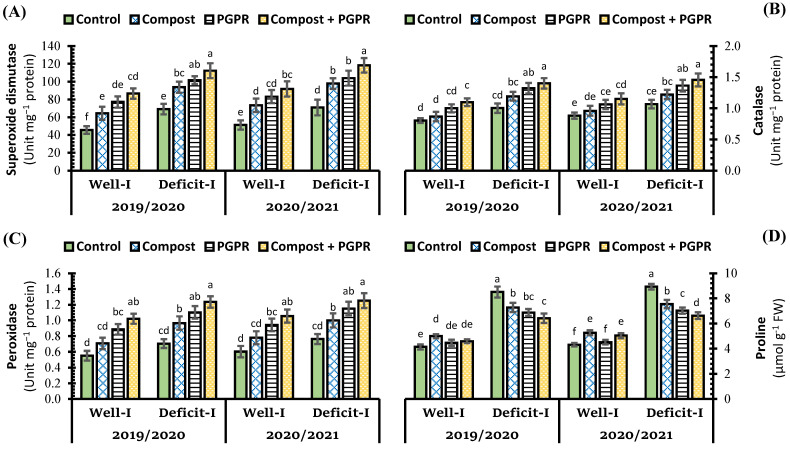
Influence of soil amendment with compost and plant growth-promoting rhizobacteria (PGPR) on the status of antioxidant system (**A**) superoxide dismutase, (**B**) catalase, (**C**) peroxidase, and (**D**) proline concentration in the leaves of wheat plants grown in salt-affected soil and deficit irrigation in contrast to well irrigation. The error bars present the values of standard deviation. According to Duncan’s multiple range test, the same lower-case letters above columns are not significantly different (*p* < 0.05).

The antioxidant enzymes superoxide dismutase (SOD), peroxidase (POD), and catalase (CAT) activities in the leaves of wheat plants growing under deficit irrigation are depicted in Figure 4 in relation to compost and/or PGPR applications. In comparison to conventional irrigation, the relationship between deficit irrigation and soil salinity resulted in increased SOD, CAT, and POD, which increased by 51.3%, 24.8%, and 27.7%, respectively. Although subjecting wheat plants to stress conditions increased the activity of non-enzymatic antioxidants, treating plants with compost or PGPR as a sole or combined application increased the activity levels of these enzymes either under regular or deficit irrigation. The application of PGPR was superior to compost application in increasing the enzyme activity, which under regular irrigation increased SOD by 68.9%, CAT by 24.5%, and POD by 60.9%, while under deficit irrigation, increased the activity of SOD by 46.8%, 32.0%, and 56.8%, respectively (Figure 4A–C). Combined application (compost + PGPR) recorded the maximum enzyme activity under all studied conditions. 

The concentration of proline as a non-enzymatic antioxidant recorded its maximum level in wheat leaves subjected to deficit irrigation (Figure 4D). Under regular irrigation, proline levels increased slightly under the application of compost and/or PGPR. Whereas, under deficit irrigation conditions, the same treatments reduced proline levels below their control (−). The best treatment in reducing proline content under deficit irrigation was the combined application, which reduced the concertation by 24.6%.

### 2.4. Effect of Soil Treatments on Yield Parameters and Related Traits under Abiotic Stress

**Figure 5:**
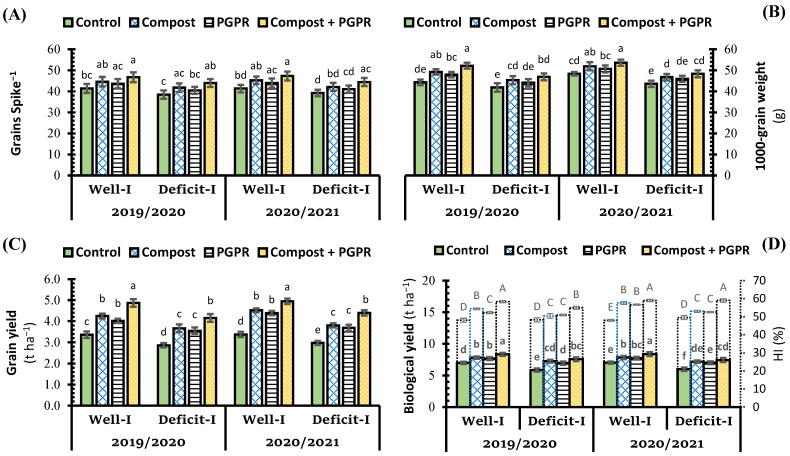
Influence of soil amendment with compost and plant growth-promoting rhizobacteria (PGPR) on the grain yield traits (**A**) grain spike^−1^, (**B**) 1000 grain weight, (**C**) grain yield, and (**D**) biological yield and harvest index (HI) of wheat plants grown in salt-affected soil and deficit irrigation in contrast to well irrigation. The error bars present the values of standard deviation. According to Duncan’s multiple range test, the same lower-case letters above columns are not significantly different (*p* < 0.05), while the upper-case letters in figure (**D**) presented the significant differences between means of HI.

Although 1000-grain weight, grains yield, and biological yield of wheat plants significantly decreased when wheat plants were subjected to deficit irrigation in salt-affected soil during growth seasons (Figure 5B–D), the number of grains spike^−1^ and harvest index (HI) were less affected than previous yield parameters (Figure 5A,D). Soil amendments either with compost or PGPR as an individual application slightly increased the number of grains spike^−1^ under both irrigation conditions. Whereas the combined application with both soil amendments increased the number of grains spike^−1^ to significant levels, which recorded an increase by 12.8% and 14.3% over both types of control (+ and −), respectively. Compost application was more effective than PGPR application in significantly increasing yield attributes, i.e., 1000-grain weight, grains yield, and HI% wheat plants (Figure 5B–D). Compost application led to an increase in the 1000-grain weight (by 11.2% and 8.3%), grains yield (by 26.3% and 28.3%), and HI% (by 13.1% and 4.5%) over both types of control (+ and −), respectively. The co-application of compost and PGPR extended their positive effects on all studied yield attributes, i.e., number of grains spike^−1^, 1000-grain weight, grains yield, biological yield, and HI% of wheat plants to record the best results. The increment percentage under the effect of combined application (compost + PGPR) recorded 18.0%, 45.0%, 19.4%, and 21.5% over control (+) for 1000-grain weight, grains yield, biological yield, and HI%, respectively. Whereas, under deficit irrigation, the increment percentages under the effect of combined application were 12.1%, 45.4%, 29.2%, and 13.9%, respectively (Figure 5B–D). 

### 2.5. Effect of Soil Treatments on Nutrient Uptake under Abiotic Stress

**Figure 6:**
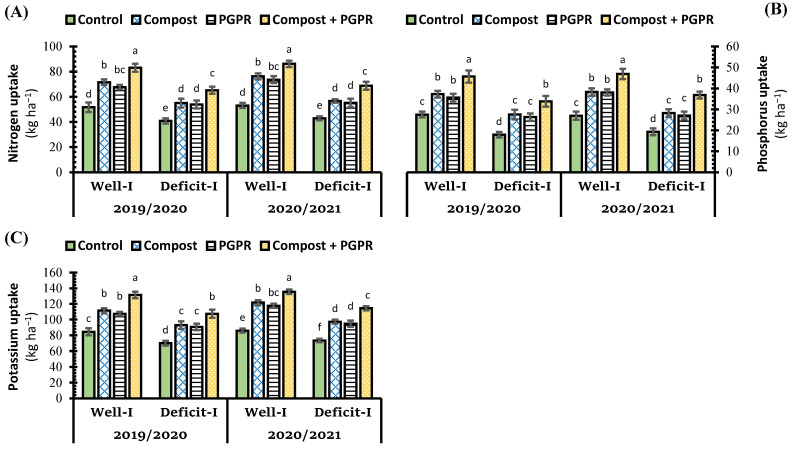
Influence of soil amendment with compost and plant growth-promoting rhizobacteria (PGPR) on the uptake of (**A**) nitrogen, (**B**) phosphorus, and (**C**) potassium by the grains of wheat plants grown in salt-affected soil and deficit irrigation in contrast to well irrigation. The error bars present the values of standard deviation. According to Duncan’s multiple range test, the same lower-case letters above columns are not significantly different (*p* < 0.05).

The ability of wheat grains to uptake N, P, and K was significantly lessened under deficit irrigation during both growing seasons (Figure 6), which reduced the uptake content of N, P, and K by 21.1%, 34.9%, and 17.1%, respectively. As the individual application with compost was better than PGPR application for yield attributes (Figure 5), the same trend was recorded for nutrient uptake (Figure 6). Under regular irrigation, compost application increased the uptake of N, P, and K by 38.4%, 35.3%, and 31.6%, respectively, while the increments under the effect of PGPR were 30.8%, 29.5%, and 27.1%, respectively. Whereas under deficit irrigation, the increments in the uptake of N, P, and K by compost application were 34.6%, 53.6, and 32.6%; meanwhile, for PGPR application, the increment percentages were 32.0%, 46.6%, and 29.3%, respectively. During the growth seasons, when wheat plants were treated with the co-application of compost and PGPR, a significant increase in grain N, P, and K uptake was observed, which mitigated the deleterious effect of deficit irrigation on wheat plants grown in salt-affected soil (Figure 6). It was found that wheat plants treated with a synergistic application of compost and PGPR absorbed more nitrogen, phosphorus, and potassium from the soil over the course of two years, regardless of irrigation type. The combined application (compost + PGPR) had significantly maximized grain N, P, and K uptake over the rest of the applications, which increased their contents under regular irrigation by 60.6%, 66.0%, and 55.4%, respectively, whereas under deficit irrigation condition the increment levels over control were 59.6%, 88.7%, and 53.3%, respectively (Figure 6).

## 3. Discussion

### 3.1. Effect of Soil Treatments on Soil Exchangeable Na Percentage, Soil Enzymes, Ion Selectivity and Photosynthetic Pigment Contents under Abiotic Stress

Water stress and soil salinity have together exacerbated a severe ecological threat for agricultural sustainable development which has caused a weakened soil structure, low aeration, surface crusting, high pH, and low filtration rate [21,38]. Moreover, osmotic stress limits water and nutrient uptake resulting in decreased soil enzymes activity and soil physicochemical properties [39]. 

From our experiment, a higher concentration of exchangeable Na than Ca and Mg under saline soil and water stress conditions together caused excessive Na content and increased ESP, which lessened soil quality, plant growth and eventually decreased crop productivity [23,40]. 

Our findings showed that coupled application of compost and PGPR significantly improved wheat growth which can relieve salt and water stress by adsorbing Na ions and releasing non-toxic beneficial Ca, Mg, and K ions which reflect positively on the water holding capacity, soil health, and plant health compared to unamended soil. Ismail et al. [41] stated that the application of compost decreased ESP and enhanced the physicochemical properties including the increment in CEC, nutrient uptake, water holding capacity, and soil pH. 

Moreover, it was stated that compost has an important impact on plant growth and development under water stress and soil salinity [42]. Compost is considered a carbon source for microbes, which can improve the organic carbon to the soil resulting in an improvement in soil physicochemical properties and soil enzymes activity [24,43]. Compost is a well-known source of micro and macronutrients for plants [43]. Compost acts as a carrier material for bacterial inoculation of cereals, thus improving their microbial activities through improved ACC-deaminase activity to combat ethylene stress, production of phytohormones, exopolysaccharides, organic acids, etc., that improves the water holding capacity and osmoregulation [44]. Therefore, the coupled application of compost and PGPR has a synergistic impact on crop growth and productivity under water stress and soil salinity.

From our study, it was found that PGPR plays a crucial role in augmenting the water holding capacity due to its characteristics in ameliorating soil hydrological properties [45], which cause increments in soil enzyme activity and improved soil quality. Soil enzymes activity (urease and dehydrogenase) was increased in response to PGPR inoculation which in turn improved root structure, soil particulates, and polysaccharides from microbial cells [23]. Consequently, an improvement in porosity, aeration, infiltration, and water uptake by roots reflecting positively on plant water status and plant ions was observed [21]. PGPR could secrete organic acids and phytohormones, acting as biocontrol agents. An increase in soil enzymes activity in response to PGPR inoculation ameliorates ethylene stress via ACC deaminase production [18].

The application of compost and PGPR triggered a cascade of reactions that stifled Na and increased Ca and Mg in soil solution resulting in a decline in the exchangeable sodium percentage in the soil which decreased Na uptake and increased K uptake into wheat leaves under soil salinity and water stress together [46]. In line with our findings, it can be stated that ion homeostasis in plants is enhanced under abiotic stress conditions after adding the combined application of compost and PGPR for wheat than individual application [47]. Therefore, the combined application of compost and PGPR seems to be applicable in plummeting Na uptake and improving plant water balance resulting in the improvement in photosynthetic pigment contents in wheat plants under abiotic stress [48].

The combined application of compost and PGPR remarkably improved plant growth through increased chlorophyll *a*, *b* and carotenoids in leaves under saline soils and water stress together which helped in cell elongation and division [49]. According to our findings, it was observed that the combined application of compost and PGPR enhanced the soil water holding capacity and plant bioavailable water; therefore, water can absorb easily resulting in increased relative water content in the plant leaves alongside osmoregulation than an individual application under water stress and soil salinity together [50]. 

### 3.2. Effect of Soil Treatments on Physiological Attributes under Abiotic Stress

The improvement in physiological features positively reflects leaf health and crop performance as well as hydraulic conductivity [51,52]. However, under water stress and soil salinity, physiological processes such as proline content, stomatal conductance, and relative water content are negatively impacted resulting in a decline in plant development [53,54]. In the current research, it was found that the application of compost not only improved soil physicochemical properties but also augmented the amount of available macro- and micro-nutrients for plant growth and physiological processes [55]. Compost application enhanced the water holding capacity (WHC) and nutrient retention which increased stomatal conductance, relative water content resulting in a decline in proline content under water stress and soil salinity [56]. It was also found that PGPR inoculation improved root hydraulic conductivity and promoted carbohydrate metabolism and transport, which is positively reflected in physiological processes [57]. Therefore, the coupled application of compost and PGPR resulted in a further improvement in physiological processes due to their synergistic benefits on root structure, plant growth, and development. 

### 3.3. Effect of Soil Treatments on Antioxidant Enzyme Activity and Oxidative Stress Indicators in Wheat Leaves under Abiotic Stress

Under water stress and soil salinity together, oxidative stress may increase in plants owing to the exacerbation of the production of reactive oxygen species (ROS) [58,59]. Under these circumstances, high H_2_O_2_ levels were observed due to their conversion into a more toxic hydroxyl anion, leading to membrane degradation and lipid peroxidation due to their extremely harmful to plant cells as a consequence of their influential oxidative nature [60]. In line with our results, it was previously reported on wheat plants under abiotic stress which resulted in severe oxidative stress to plants [61]. From our results, it was shown that the individual application of compost or PGPR under abiotic stress (water stress and soil salinity) retained the redox balance of the cell and resulted in a notable reduction in H_2_O_2_ contents and a decrease in lipid peroxidation (MDA) and electrolyte leakage compared to unamended treatment (control) [62]. However, the coupled application of compost and PGPR was more influential in amending oxidative damage than the sole application resulted in transforming ROS into less or non-toxic compounds [63]. This is a clear indication of the ameliorative role of coupled application of compost and PGPR in decreasing oxidative damage in wheat plants subjected to water stress and soil salinity together.

In the current investigation, the antioxidant enzymatic activities, such as SOD, CAT, and POX, were augmented to a certain level in wheat plants under water stress and soil salinity together. Nevertheless, this increment was not enough to detoxify the harmful impacts of reactive oxygen species. Conversely, the sole application of compost caused a noticeable augment in SOD, CAT, and POX activities [64]. However, it was noted that SOD, CAT, and POX activities are further increased under the coupled application of compost and PGPR perhaps owing to reduced Na uptake [65]. In harmony with our findings, prior investigations presented augmented SOD, CAT, and POX under abiotic stress [66]. These enzymes convert H_2_O_2_ into non-toxic compounds such as H_2_O and O_2_ and thus protect the plants from their harmful impacts on cell membranes and macromolecules [67]. Curiously, the activities of these enzymes were further augmented due to the coupled application of compost and PGPR under water stress and soil salinity together [8]. The results of this investigation encourage the hypothesis that the coupled application of compost and PGPR increases SOD, CAT, and POX activities in wheat plants exposed to abiotic stress which positively reflects wheat growth and grain yield and decreases oxidative damage [18].

### 3.4. Effect of Soil Treatments on Yield Parameters and Related Traits under Abiotic Stress

Our findings showed that yield-related traits and cop production including the number of grains spike^−1^, 1000-grain weight, grain yield, biological yield, and harvest index were significantly reduced in wheat plants under water stress and soil salinity together. However, the combined application of compost and PGPR augmented these parameters compared to the sole application. Under salt and water stress, the combined application of compost and PGPR has been shown to increment RWC and chlorophyll contents in wheat leaves owing to an improvement in water retention, soil fertility, and a decline in the bulk density, causing an observed improvement in plant water balance resulting in higher growth, grain yield and its attributes [8,45].

### 3.5. Effect of Soil Treatments on Nutrient Uptake under Abiotic Stress

Soil salinity individually or/and water stress deleteriously influenced grain N, P, and K uptake. PGPR inoculation could produce phytohormones, organic acid, IAA, phosphatases, gibberellins, and minerals that ultimately improved soil nutrient cycling and the water holding capacity [7,29]. Our inocula, containing Bacillus, plays an imperative role as a growth promoter, comates the water stress and salt stress, and improves water and nutrient uptake, which eventually increases N, P, and K uptake; our inocula also contained Azospirillum which has the potential to biological nitrogen fixation (BNF) through a symbiotic relationship between Rhizobia and plants which resulted in a higher grain N, P, and K uptake under abiotic stresses [30,31]. However, compost application as a soil conditioner had a crucial impact on the formation and stabilization of soil aggregates and improved the soil’s cation exchange capacity, and increased the holding of nutrients resulting in augmenting grain N, P, and K uptake under water stress and soil salinity [39]. Our findings stated that coupled compost and PGPR had a higher N, P, and K uptake than the sole application due to their synergistic impact on physiological processes as well as crop productivity and nutrient uptake.

## 4. Materials and Methods

### 4.1. Study Site Description and Plant Material

Two field experiments were investigated at the Elamaar village in the region of Sidi Salem (31°07 N latitude, 30°57 E longitude), Kafr El-sheik Governorate, Egypt, during the 2019/2020–2020/2021 cropping seasons, to investigate the application of compost as a soil amendment coupled with PGPR inoculation on soil properties, soil enzymes, growth, physiological, and nutrient uptake as well as yield traits and productivity of wheat (*Triticum aestivum*) under water stress in salt-affected soil. Wheat seeds (cv; Misr 1; 140 kg ha^−1^ sowing rate) were introduced from the Wheat Research Program, Field Crops Research Institute, Egypt. Wheat grains were planted on the 15th and 18th of November for both years of study, respectively. Soil samples were collected at depth (0–30 cm) before sowing to measure the physical and chemical characteristics in both seasons. The experimental soil was analyzed and its physical and chemical properties were as follows; soil texture was clay loam and the chemical properties, respectively, in both seasons were pH (8.35 and 8.58), EC (8.14 and 9.09 dS m^−1^), O.M. (1.51 and 1.48%), field capacity (29.4 and 31.1%), Na^+^ (17.78 and 18.65 meq L^−1^), K^+^ (10.73 and 11.86 meq L^−1^), Mg^+2^ (14.76 and 16.23 meq L^−1^), Ca^+2^ (6.54 and 18.29 meq L^−1^), HCO_3_^−^(17.22 and 19.67 meq L^−1^), and SO_4_^−2^ (19.03 and 21.15 meq L^−1^). Meteorological data of the experimental site are also presented in Table 3.

### 4.2. Experimental Design and Treatments

The field experiment design was arranged in a split-plot at three replicates comprising eight treatment combinations, i.e., two water irrigation regimes as follows: well irrigation (four irrigations) and deficit irrigation (two irrigations) in the main plots and four soil treatments in subplots. Four treatments comprised of (1) non-treated control, without the application of compost or PGPR; (2) soil application of compost; (3) inoculation with PGPR; (4) combined application of compost and PGPR. The subplot net area was 10.50 m^2^ (3 × 3.5 m) with a plant spacing of 15 cm. The research experiments were carried out in accordance with the most important technical recommendations contained in the Bulletin of the Ministry of Agriculture and Land Reclamation. The soil of the experimental plot was prepared in the same manner as the nursery area. Prior to wheat planting, ploughing was performed at 30 cm depth and hoeing at 15 cm during soil preparation. The experimental soil was fertilized with phosphorus fertilizer in the form of calcium superphosphate (15.5% P_2_O_5_) at a rate of 107 kg P_2_O_5_ ha^−1^ during land preparation and nitrogen fertilizer was applied at a rate of 286 kg ha^−1^ as ammonium nitrate (33.5%). Nitrogen fertilizer was added in two portions, half being applied before the first irrigation, while the remaining portion was applied before the second irrigation.

#### 4.2.1. Compost Characterization

Crop residues (rice, cotton, and maize straw) were used as materials of compost. After complete decomposition, the compost was applied to the soil at a rate of 12 tons ha^−1^. Compost analysis showed that the main characteristics of our substrate were: organic matter (40%), EC (2.9 dS m^−1^), total N (1.9%), total P (9.7%), total K (0.44%), and pH (7.5).

#### 4.2.2. Microorganisms and Culture Conditions

Bacterial inoculants of plant growth-promoting rhizobacteria (PGPR), namely *Azospirillum lipoferum* SP2, *Bacillus coagulans* NCAIM B.01123, *Bacillus circulance* NCAIM B.02324, and *Bacillus subtilis* MF497446, were attained from the Bacteriology Laboratory, Sakha Agricultural Research Station, Kafr El-Sheikh, Egypt. The standard culture circumstances were set with a semi-solid malate medium for *A. lipoferum* [68], and with a nutrient broth medium for the *Bacillus* strains [68]. Total counts of microbes were 56 ± 2.23 CFU × 10^5^ g^−1^ dry soil for *Bacillus* and 37 ± 1.67 CFU × 10^5^ g^−1^ dry soil for *Azospirillum*. Total counts were performed by pasteurization in a water bath at 70 °C for 10 min to kill the vegetative cells. The inoculation treatments were prepared as peat-based inoculums, with 30 mL of 10^9^ CFU mL^−1^ from each culture per 60 g of sterilized carrier. A 1.5 kg sterilized carrier included (1:1:1:1) *A. lipoferum*, *B. coagulans*, *B. circulance*, and *B. subtilis* which were mixed with 140 kg ha^−^^1^ wheat seeds before sowing.

### 4.3. Soil Measurements

#### 4.3.1. Soil Exchangeable Percentage of Na^+^

At harvesting time of wheat, soil samples were collected from every treatment 0–30 cm soil depth by an auger. Soil samples were oven-dried and passed through a 2 mm net to evaluate soil Na^+^, Ca^2+^, and Mg^2+^ contents in paste extract by atomic absorption spectrophotometer (AAS, PERKIN ELMER 3300) to calculate the soil sodium adsorption ratio (SAR). Then, ESP was estimated based on the equation proposed by Arshad et al. [69]:

$$\mathrm{ESP}\;=1.95+1.03\times \;\mathrm{SAR}\;\left(R^{2}=0.92\right)$$

#### 4.3.2. Soil Enzymes (Dehydrogenase and Urease) Activity

At 75 days after planting, soil samples were selected at 0–30 cm depth to determine soil enzymes (dehydrogenase and urease) activity. The selected soil samples were cleansed carefully bypassed across a 5 mm mesh to eliminate plant residues and large particles, such as fragments, and then maintained in polyethylene bags at −20 °C for additional analysis. Urease enzyme activity was assayed according to the quantitative determination of ammonia by the spectrophotometric measurement at 660 nm [70]. In addition, the Mersi method [71] was used to determine the dehydrogenase enzyme activity by applying the selected soil samples on INTsolution, keeping them for 2 h at 40 °C. The reduced iodonitro-tetrazolium formazan (INTF) was extracted with dimethyl-formamide and ethanol then calculated photometrically at 464 nm.

### 4.4. Plant Measurements

#### 4.4.1. Measurements of Na^+^ and K^+^ Ions in Leaves 

Leaf strip (1 g) was selected from fully expanded leaves at 75 days after sowing and digested with 8 mL of digestion mixture HNO_3_:HClO_4_ (3:1 *V/V*) based on the method by Sparks et al. [72] at 75 days after sowing to measure the Na^+^ and K^+^ contents as mg g^−1^ dry weight.

#### 4.4.2. Physiological Attributes

##### Photosynthetic Pigment Contents 

Leaf strip (1 g) was selected from fully expanded leaves at 75 days after sowing was extracted overnight with 80% acetone (5 mL), then grounded and homogenized. The homogenized mixtures were filtered, and the filtrates were raised to 25 mL by adding 80% acetone in each sample. The absorbance of the filtrates was determined at various wavelengths (663, 645, and 480 nm) using a UV-1900 BMS (Waltham, MA, USA) spectrophotometer. All readings were taken for three samples from each replicate per treatment. The concentrations were calculated according to the method by Lichtenthaler [73]. The equations used for calculations are as follow:

Chl *a* (mg g^−1^ FW) = 12.7 (A_663_) − 2.69 (A_645_)

Chl *b* (mg g^−1^ FW) = 25.8 (A_645_) − 4.68 (A_663_) 

Carotenoids (mg g^−1^ FW) = (1000 (A_470_) − 2.27 (Chl *a*) − 81.4 (Chl *b*))/227

##### Stomatal Conductance

Stomatal conductance (g_s_) was determined on fully expanded flag leaves from the adaxial and abaxial surfaces from five plants in each experimental unit with a dynamic diffusion porometer (Delta-T AP4, Delta-T Devices Ltd., Cambridge, UK) were obtained at fine days according to Izanloo method [74]. Measurements were recorded from the central area of front (r_a_) and back side (r_b_) of the top full expanded leaf. Total leaf conductance (r_l_) is 1∕r_l_ = 1∕r_a_ + 1∕r_b_ as mmol H_2_O m^−2^ s^−1^.

##### Leaf Water Relations

Fresh leaf samples (0.5 g) (FW) were soaked in water until constant weight. The saturated leaves were weighed (TW) and then dried for 24 h at 80 °C for estimation of dry weight (DW). Leaf relative water content (RWC%) was calculated based on the described methods by Weatherley [75].

$$\mathrm{Relative}\;\mathrm{water}\;\mathrm{content}\;\left(\mathrm{RWC}\right)=\frac{\left(\mathrm{FW}\;-\mathrm{DW}\right)}{\left(\mathrm{TW}\;-\mathrm{DW}\right)}\times 100$$

#### 4.4.3. Oxidative Stress Indicators and Antioxidant System in Wheat Leaves

##### Oxidative Stress Indicators 

Leaf H_2_O_2_ was estimated following the method projected by Velikova et al. [76]. The procedure was carried out by homogenization of sample (0.5 g) using liquid N_2_ and trichloroacetic acid (TCA: 0.1%). It was followed by centrifugation of the homogenized sample at 3000 rpm for 20 min. Then, the analysis mixture was set by using 10 mM K-phosphate buffer (pH 7.0, 1.0 mL), potassium iodide (2 M, 1 mL), and plant extract (1 mL), and then the mixture absorbance was recorded at 390 nm by a spectrophotometer. A standard curve was also drawn under identical conditions and H_2_O_2_ contents were calculated as µmol g^−1^ FW. 

Lipid peroxidation (MDA) in terms of thiobarbituric acid reactive substances (TBARS) was assessed based on the method illustrated by Du and Bramlage [77]. About 0.5 g leaf segments were ground in liquid N_2_ and hydro-acetone buffer (4:1 *v*/*v*). Then, 0.65% thiobarbituric acid (TBA) and 0.01% butyl hydroxyl toluene (BHT) were applied and samples were incubated at 95 °C. After incubation, the mixture was exposed to centrifugation at 10,000× *g* for 15 min. The mixture absorbance was measured spectrophotometrically at 532 and 600 nm which was expressed in nmol g^−1^ FW.

Electrolyte leakage (%) was determined in the topmost fully expanded leaves at 75 days after sowing. Ten discs (1 cm^2^) were immediately weighed and cut into segments which were then placed into a test tube including 25 mL of distilled water. The test tubes were shaken (100 rpm) at room temperature for 24 h to facilitate electrolyte leakage from tissues. Initial electrical conductivity measurements were calculated. Flasks were then immersed in a hot water bath (80 °C) for 1 h to induce cell rupture. The samples were again placed on the shaker for 20 h at 21 °C. Final conductivity was measured for each flask. The percentage of electrolyte leakage (*EL*; %) was measured by the following formula [78]:

$$EL=\frac{\left(Initial\;conductivity\right)}{\left(Final\;conductivity\right)}\times 100$$

##### Antioxidant System

Leaf segments of wheat (250 mg each) were crushed and homogenized in 5 mL of cold phosphate buffer (50 mM phosphate buffer pH 7.0, containing 1 mM EDTA, 1 mM phenylmethylsulfonyl fluoride, and 1% polyvinylpolypirrolidone) to use as an enzyme extract [59]. Later, the homogenized sample was centrifuged at 15,000× *g*, 4 °C for 30 min. The activity of superoxide dismutase (SOD: 1.15.1.1) was determined based on a 50% NBT reduction assay at 560 nm as described by Beauchamp and Fridovich [79]. The activity of catalase (CAT: 1.11.1.6) was performed respecting the reaction between 50 µL of enzyme extract and 12.5 mm of H_2_O_2_ in the presence of 50 mM of K-phosphate buffer (pH 7.0). The reaction was started by adding H_2_O_2_ and the absorbance was monitored at 240 nm for 60 s [80]. Peroxidase (POX: 1.11.1.7) activity was determined using o-phenylenediamine as a chromogenic indicator in the presence of H_2_O_2_ and enzyme extract at 417 nm as described by Vetter et al. [81]. The activity of all enzymes was presented as units mg^−1^ protein.

Proline content was estimated based on the method by Bates et al. [82]. Five fully expanded leaves with roughly 0.5 g of each experimental unit were mixed smoothly in 5 mL of 3% sulfosalicylic acid. Ninhydrin reagent (2 mL) and glacial acetic acid (2 mL) were applied to the test tube with 2 mL of extract. The combination was subjected to an oven at 90 °C for 30 min. The reaction was put in an ice bath. Therefore, 5 mL of toluene was applied to the reaction combination once cooled and vortexed, combined for 15 s, and maintained in the dark for 20 min at room temperature to permit separation of the toluene layer from the aqueous solution. Each toluene layer was then cautiously obtained into a clean tube, and absorbance was read at 520 nm by a UV-1900 BMS spectrophotometer. Free proline content was estimated from a standard curve prepared by analytical grade proline and expressed as mg g^−1^ FW.

### 4.5. Yield Parameters and Related Traits

After the final harvest, ten wheat plants from each experimental unit were randomly collected for calculating the number of grains spike^−1^ and 1000-grain weight (g). At the same time, a 6 m^2^ area was harvested from the middle of each plot to measure grain and straw yield. The biological yield was calculated by straw and grain yield taking into consideration grain moisture (14%). The harvest index (%) was computed using the equation:

$$\mathrm{Harvest}\;\mathrm{index}=\frac{\mathrm{Grain}\;\mathrm{yield}\;\left({t\;\mathrm{ha}}^{-1}\right)}{\mathrm{Biological}\;\mathrm{yield}\;\left({t\;\mathrm{ha}}^{-1}\right)}\times 100$$

#### Nutrient Uptake

After the final harvest, ten wheat plants from each experimental unit were randomly collected and separated into grain and straw. Air-dried grain samples were placed into a forced-air oven for 48 h at 70 °C. Then, the grain samples were powdered using a grinder for determining grain N, P, and K uptake. Nitrogen, phosphorus, and potassium contents in wheat grain were determined by micro Kjeldahl’s according to the procedure of A.O.A.C. [83], spectrophotometer, and flame photometer according to the procedure of Sparks et al. [72], respectively.

### 4.6. Statistical Analysis

All the values were expressed as the mean ± standard deviation (S.D.) and analyzed for ANOVA and post hoc Duncan’s *t*-test. Differences between groups were considered significant at *p* < 0.001 and *p* < 0.05 levels.

## 5. Conclusions

Soil health, plant development, grain yield, and nutrient uptake of wheat declined considerably under water stress and soil salinity together in the absence of soil amendments. However, the application of compost and plant growth-promoting rhizobacteria separately or in combination under water stress alongside salt-affected soil increased the plant growth and grain yield of wheat. The coupled application was shown to be more efficient in alleviating soil ESP by decreasing the accumulation of Na and reducing oxidative damage. The amendment of soil salinity with compost and PGPR appears to be a very promising technique for augmenting crop yields and sustainable agriculture under water stress and soil salinity.

## Figures and Tables

**Table 1:** Influence of soil amendment with compost and plant growth-promoting rhizobacteria (PGPR) on the exchangeable Na percentage (ESP), and the activity of urease and dehydrogenase in the soil cultivated with wheat plants and subjected to deficit irrigation in contrast to well irrigation.

Irrigation	Soil Amendments	ESP (%)		Urease (mg N-NH_4_^+^ g^−1^ soil h^−1^)		Dehydrogenase (mg TPF g^−1^ DM soil d^−1^)	
		2019/2020	2020/2021	2019/2020	2020/2021	2019/2020	2020/2021
Well Irrigation	Control	18.0 ± 0.4 c	16.5 ± 1.2 b	115.9 ± 4.1 e	124.0 ± 5.5 d	59.3 ± 4.7 e	62.9 ± 3.1 f
	Compost	11.9 ± 0.6 f	11.4 ± 0.4 d	149.3 ± 6.2 d	158.8 ± 5.0 c	90.8 ± 3.7 cd	96.0 ± 4.0 d
	PGPR	14.8 ± 0.6 e	13.1 ± 1.0 cd	167.1 ± 4.6 b	181.9 ± 5.1 b	110.9 ± 5.3 b	116.7 ± 4.7 b
	Compost + PGPR	9.6 ± 1.0 g	9.1 ± 1.0 e	214.4 ± 6.0 a	223.4 ± 7.2 a	130.4 ± 5.5 a	134.6 ± 5.3 a
Deficit Irrigation	Control	23.8 ± 0.7 a	22.4 ± 0.6 a	85.8 ± 3.2 g	94.7 ± 3.4 e	36.6 ± 3.1 f	48.5 ± 4.0 g
	Compost	18.2 ± 1.2 c	17.4 ± 0.4 b	109.0 ± 4.0 f	116.6 ± 5.2 d	69.3 ± 2.6 e	79.8 ± 5.1 e
	PGPR	21.2 ± 1.3 b	20.7 ± 1.0 a	118.8 ± 5.1 e	122.8 ± 4.7 d	83.6 ± 3.1 d	92.6 ± 4.3 d
	Compost + PGPR	16.6 ± 0.9 d	14.3 ± 0.5 c	153.0 ± 4.9 c	160.3 ± 5.6 c	101.4 ± 4.1 bc	103.7 ± 4.2 c
Irrigation		**	***	***	**	**	**
Soil amendments treatments		***	***	***	***	***	***
Irrigation × treatments		*	*	***	**	*	*

According to Duncan’s multiple range test (*p* < 0.05), the means followed by different lower-case letters indicate significant differences among treatments. Values are presented as the means ± standard deviation (SD). ***, **, and * denote significance at *p* < 0.001, <0.01, and <0.05 respectively.

**Table 2:** Influence of soil amendment with compost and plant growth-promoting rhizobacteria (PGPR) on the content of sodium (Na) and potassium (K) in the leaves of wheat plants grown in salt-affected soil and deficit irrigation in contrast to well irrigation.

Irrigation	Soil Amendments	Na (mg g^−1^ DW)		K (mg g^−1^ DW)		K/Na (Ratio)	
		2019/2020	2020/2021	2019/2020	2020/2021	2019/2020	2020/2021
Well Irrigation	Control	4.24 ± 0.19 d	4.30 ± 0.33 d	3.04 ± 0.08 de	3.10 ± 0.09 e	0.72 ± 0.05 de	0.72 ± 0.06 d
	Compost	3.37 ± 0.14 f	3.45 ± 0.08 f	3.87 ± 0.10 b	3.88 ± 0.13 b	1.15 ± 0.03 b	1.13 ± 0.06 b
	PGPR	3.68 ± 0.21 e	3.75 ± 0.07 e	3.56 ± 0.11 c	3.65 ± 0.12 c	0.97 ± 0.06 c	0.98 ± 0.05 c
	Compost + PGPR	3.02 ± 0.19 g	3.02 ± 0.14 g	4.28 ± 0.18 a	4.40 ± 0.10 a	1.42 ± 0.15 a	1.46 ± 0.09 a
Deficit Irrigation	Control	6.16 ± 0.30 a	6.01 ± 0.21 a	2.28 ± 0.11 g	2.38 ± 0.13 g	0.37 ± 0.01 g	0.04 ± 0.03 f
	Compost	4.73 ± 0.15 c	4.74 ± 0.20 c	2.9 ± 0.09 ef	2.98 ± 0.08 ef	0.61 ± 0.03 ef	0.63 ± 0.02 e
	PGPR	5.11 ± 0.15 b	5.19 ± 0.14 b	2.67 ± 0.10 f	2.83 ± 0.10 f	0.52 ± 0.04 f	0.54 ± 0.01 e
	Compost + PGPR	4.17 ± 0.16 d	4.25 ± 0.18 d	3.31 ± 0.13 cd	3.43 ± 0.11 d	0.79 ± 0.03 d	0.81 ± 0.05 d
Irrigation		**	**	**	**	**	**
Soil amendments treatments		***	***	***	***	***	***
Irrigation × Treatments		*	*	ns	ns	**	**

According to Duncan’s multiple range test (*p* < 0.05), the means followed by different lower-case letters indicate significant differences among treatments. Values are presented as the means ± standard deviation (SD). ***, **, *, and ns denote significance at *p* < 0.001, <0.01, <0.05 and non-significant, respectively.

**Table 3:** Meteorological data of the experimental sites between 2019/2020 and 2020/2021 growing seasons.

Year Month	2019/2020				2020/2021			
	Temperature (°C)		Rainfall	Relative Humidity (%)	Temperature (°C)		Rainfall	Relative Humidity (%)
	Max	Min	(mm)		Max	Min	(mm)	
Nov	26.3	17.2	0.98	32.6	25.3	16.2	0.94	31.6
Dec	25.9	15.3	0.85	34.2	24.9	14.3	0.82	33.2
Jan	24.5	13.2	1.1	35.1	23.2	12.4	0.54	32.7
Feb	22.3	10.3	3.1	46.2	20.3	11.1	3.32	42.4
Mar	21.4	9.7	6.4	44.3	20.6	10.7	6.85	43.1
April	23.7	13.8	0.5	43.8	22.5	12.5	0.63	44.8

## Data Availability

Not applicable.
